# Ytterbium–Zinc Oxide Embedded on Hexagonal Boron Nitride Nanocomposite for Electro‐Oxidation of Ciprofloxacin

**DOI:** 10.1002/open.202500246

**Published:** 2025-06-26

**Authors:** Collen Nepfumbada, Christopher P. Woodley, Bhekie B. Mamba, Bart M. Bartlett, Usisipho Feleni

**Affiliations:** ^1^ College of Science, Engineering and Technology (CSET) University of South Africa (UNISA) Institute for nanotechnology and water sustainability (iNanoWS) Florida campus Johannesburg 1709 South Africa; ^2^ Department of Chemistry University of Michigan Ann Arbor Michigan 48109‐1055 United States

**Keywords:** ciprofloxacin, detections, electrocatalysts, metal oxides, selectivity and sensitivities

## Abstract

Metal oxide‐based electrocatalysts have become increasingly popular in the electrochemical field due to their outstanding electrocatalytic properties. Herein, a hydrothermal approach is used to synthesize the composite of ytterbium zinc oxide endorsed on 2D hexagonal boron nitride (YbZnO@h‐BN). Different analytical methods, including Fourier transform infrared (FTIR), Raman spectroscopy, field emission scanning electron microscopy (FESEM), transmission electron microscopy (TEM), and X‐ray diffraction (XRD), along with X‐ray photoelectron spectroscopy (XPS), are used to interrogate the morphological and structural characteristics of the nanocomposite. FTIR analysis reveals Zn–O and Yb–O functionality, while SEM images shows the YbZnO@h‐BN microspherical structure. Cyclic voltammetry (CV) and square‐wave voltammetry (SWV) techniques are employed for the electrochemical analysis of Ciprofloxacin (CIP) on the YbZnO@h‐BN/glassy carbon electrode (GCE)‐modified electrode. The YbZnO@h‐BN/GCE sensor demonstrates a broad linear range (0.05 μM to 100 μM) with a lower limit of detection (0.059 μM) and high sensitivity of 7.4441 μA μM^−1^ cm^−2^ under optimal conditions. Additionally, the sensor shows good responsiveness for CIP, along with notable reproducibility, stability, and selectivity. Furthermore, the genuine real wastewater sample and commercial CIP tablet are utilized to investigate the usefulness of the proposed YbZnO@h‐BN/GCE sensor for CIP detection in real practice, revealing satisfactory recovery values of 94 to 105% and 95 to 112%, respectively.

## Graphical abstract

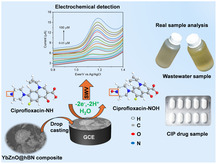

## 1 Introduction

Pharmacological compounds like antibiotics are among the most significant classes, extensively used to treat and prevent a variety of infections to both humans and animals. Ciprofloxacin (CIP), a typical synthetic second‐generation fluoroquinolone (FQ) antibiotic, is essentially used for bacterial infections triggered by both Gram‐positive and Gram‐negative broad spectrum.^[^ ^1^, ^2^ ^]^ The extensive use of CIP contributes to the occurrence and spread of resistant strains, reducing its potential therapeutic application against living organisms and creating a major threat to public health.^[^ ^3^, ^4^, ^5^, ^6^ ^]^ Hence, measuring the CIP concentration in aquatic environments is significant in addressing the ecosystem safety along with human health.^[^ ^7^ ^]^ Therefore, as environmental concerns and water safety become increasingly critical, having reliable methods to accurately measure the presence and concentration levels of CIP in water sources is of utmost importance.

Various conventional approaches, such as colorimetric, high‐performance liquid chromatography,^[^ ^8^ ^]^ spectrophotometric techniques,^[^ ^9^, ^10^ ^]^ and capillary electrophoresis,^[^ ^11^, ^12^ ^]^ among others, have been extensively explored for CIP detection. Despite their sensitivity, these methods have notable drawbacks, including massive instrument size, high cost, and the need for multiple pretreatment procedures.^[^ ^13^ ^]^ As a result, there is a continuous need for the development of an alternative strategy to quantitatively analyze antibiotic compounds in food and water samples. Electroanalytical methods have considerably drawn attention because of their potential benefits that include ease of use, portability, high selectivity and sensitivity, low cost, rapid response, and miniaturization.^[^ ^14^ ^]^ These characteristics make them promising alternatives to traditional approaches for both quantitative and qualitative analyses. Significant research has focused on developing electrochemical sensors for detecting antibiotic drugs into the environment.^[^ ^15^ ^]^ However, modifying the working electrode (WE) with an appropriate material is crucial to increase the electrode conductivity.

Metal oxide semiconductors (MOSs) have attracted attention as cost‐effective electrocatalysts in electrochemical applications, owing to their electrical and optical characteristics.^[^ ^16^ ^]^ Modification of a WE surface using MOSs is a recent and highly effective approach in the electrochemical field.^[^ ^17^, ^18^ ^]^ Among these, zinc oxide nanoparticles (ZnO NPs), especially those in the wurtzite crystal phase, are promising due to their n‐type conductivity and desirable attributes such as low cost, high electron mobility, nontoxicity, large surface area, and compatibility with doping.^[^ ^19^, ^20^ ^]^ However, challenges like low conductivity and instability prevent them from reaching their full potential. To enhance ZnO NPs electrocatalytic properties, strategies like surface modifications and doping with metallic nanoparticles have been used successfully.^[^ ^1^, ^21^ ^]^

Rare earth metals have attracted a lot of interest as dopants for a range of applications because of their luminescent, magnetic, conductive, and electrochemical character. It has been demonstrated that doping ZnO NPs with trivalent lanthanide ions such as La, Ce, Lu, Tb, Yb, and Gd, along with their oxides improves electron transfer kinetics and electrochemical performance.^[^ ^22^, ^23^, ^24^, ^25^ ^]^ Ytterbium (Yb^3+^) is known for its high optical band gap (5 eV) and ionic radius (0.89 Å), which allows it to easily replace Zn^2+^ in the ZnO crystal structure. Although YbZnO NPs have the potential to significantly improve the electrochemical properties, their full potential may not be realized when used alone as an electrode modifier. Hence, to enhance its performance, numerous materials have been incorporated as MOS support during sensor development. Recent studies have shown that materials like graphene (GR),^[^ ^26^, ^27^ ^]^ carbon nanotubes (CNTs),^[^ ^28^ ^]^ graphene oxide,^[^ ^29^, ^30^ ^]^ and hexagonal boron nitride^[^ ^31^, ^32^ ^]^ have proven to be an efficient catalyst boost for sensing platforms, owing to their distinctive characteristics that enhance the electron transfer kinetics. While GR is well‐established, h‐BN is gaining interest due to its high oxidation resistance, chemical stability, low cytotoxicity, and good mechanical strength.^[^ ^33^, ^34^ ^]^ Besides, numerous studies have shown that incorporation of h‐BN greatly enhances electrochemical performance.^[^ ^35^, ^36^, ^37^ ^]^ For this reason, integrating YbZnO NPs into h‐BN establishes a way to produce nanocomposites with highly effective sensing abilities.

Thus, a novel YbZnO@h‐BN composite was prepared using a hydrothermal system approach and used as an electrocatalyst for CIP detection. To the best of the authors’ knowledge, there is rare information regarding YbZnO@h‐BN nanocomposite in literature. The YbZnO@h‐BN/GCE sensor demonstrated high sensitivity and selectivity, good stability, a lower detection limit (LOD), and broad linearity along with excellent recoveries in real samples.

## 2 Experimental Section

### 2.1 Chemical and Solution

CIP (C_1__7_H_1__8_FN_3_O_3_, 98.0%), sulfamethoxazole (C_10_H_11_N_3_O_3_S, 98.0%), carbamazepine (C_15_H_12_N_2_O, 98.0%), naproxen (CH_3_OC_10_H_6_CH(CH_3_)CO_2_H, 98.0%), efavirenz (C_14_H_9_ClF_3_NO_2_, 98.0%), trimethoprim (C_14_H_18_N_4_O_3_, 98.5%), ampicillin (C_16_H_19_N_3_O_4_S, 96.0%), nevirapine (C_15_H_14_N_4_O, 98.0%), ytterbium(III) nitrate pentahydrate (Yb(NO_3_)_2_•5H2O, 99.9%), sodium hydroxide (NaOH), boron nitride powder (≈1 μm, 98%), hexacyanoferrate(III) ([Fe(CN)_6_]^3−/4−^ ion, zinc nitrate hexahydrate (Zn(NO_3_)_2_•6H_2_O, 99.9%), ethanol (C_2_H_5_OH, 99.9%), sodium dihydrogen phosphate monohydrate (H_2_NaPO_4_•H_2_O, 99.9%), and dibasic sodium phosphate heptahydrate (Na_2_HPO_4_•7H_2_O, 98.0%) were all acquired from Sigma Aldrich, South Africa. Chemical reagents and solvents of analytical grade were used with no additional refinement. Deionized water (DI) was used in the preparation of the buffer solution, pH correction, and nanoparticles.

### 2.2 Instrumentation

X‐ray diffraction (XRD) pattern was collected on a Rigaku SmartLab X‐ray Diffractometer fitted with a 1.5418 Å Cu Kα X‐ray radiation wavelength, 45 kV voltage, and a current of 200 mA. Raman shifts were recorded on a WITech CRM200 confocal Raman microscopy system using a 552 nm laser. The morphology and elemental composition of the samples were examined using a JEOL‐7800FLV field emission scanning electron microscopy (FESEM), coupled with energy‐dispersive X‐ray spectrometry (EDX) operated on an Oxford XMaxN 80 mm^2^ silicon‐drift, energy‐dispersive X‐ray spectrometer using Oxford Aztec v3.3 EDX acquisition and processing software for analysis. Transmission electron microscopy (TEM) was conducted using a Shimadzu JEM‐1200 EX with an accelerating voltage of 100 kV, and high‐angle‐annular dark‐field–scanning transmission electron microscopy (HAADF–STEM) was carried out using a Thermo Fisher Scientific Spectra 300 probe‐corrected STEM operated at 300 kV and acquired with a collection range of 63–200 mrad. A Dual‐X EDX system was used to do EDX mapping. The particles for HAADF–STEM analysis were dispersed in isopropanol and then drop cast onto a 300 mesh Cu TEM grid. An X‐ray photoelectron spectroscopy (XPS) collected on a Kratos Axis Supra+ with a monochromatic Al‐Kα excitation source and a pass energy of 160 eV for survey scans and 20 eV for elemental scans was used to further investigate the chemical composition. The C(1s) peak was used to reference the spectra to 284.8 eV in order to adjust for charge. Every peak was fitted using the Tougaard‐type background in Casa XPS.^[^ ^38^ ^]^ Using Fourier transform infrared (FTIR) spectroscopy (Perkin Elmer spectrum 100 FTIR, Waltham), infrared spectral analysis was performed to ascertain the chemical structure of the prepared materials in KBr pellet mode approach and recorded in the 400 to 4000 cm^−1^ range. A biologic potentiostatic mode technique (Sp50e, Autolab) was used to conduct the electrochemical investigations. Using a typical three‐electrode setup, a platinum wire, a glassy carbon electrode (GCE), and Ag/AgCl (saturated NaCl) reference electrode, square‐wave voltammetry (SWV), electrochemical impedance spectroscopy (EIS), and cyclic voltammetry (CV) were used to evaluate the electrochemical characteristics of modified WEs.

### 2.3 Preparation of ZnO NPs

**Scheme 1:**
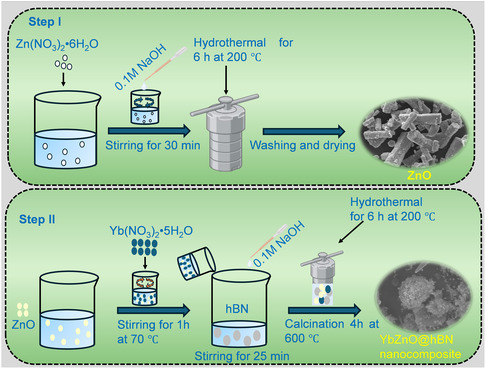
Synthesis route of ZnO (Step I) and YbZnO@h‐BN (Step II).

The ZnO NPs were prepared using a hydrothermal approach with slight modifications.^[^ ^39^ ^]^ In this modified method (**Scheme** **1** Step I), about 2 g of Zn(NO_3_)_2_•6H_2_O was dissolved in 30 mL of DI and stirred magnetically for 30 min. Next, 20 mL of ethanol solution was added while being stirred. This was followed by the dropwise addition of 0.1 m NaOH to the above mixture until the pH reached 10, and stirring continued for 1 h. Finally, the solution was then placed into a 100 mL stainless steel autoclave reactor (filled to 70%) and heated at 200 °C for 6 h. After cooling, the product was centrifuged, washed three times with absolute ethanol and DI water, and left to dry overnight at 70 °C.

### 2.4 Preparation of YbZnO@h‐BN Nanocomposite

The YbZnO@h‐BN composite was prepared following a one‐pot hydrothermal method (Scheme 1 Step II) that allowed YbZnO NPs to attach to the 2D hexagonal boron nitride (h‐BN) matrix. Initially, 0.0242 g of h‐BN was weighed in 20 mL of isopropanol and agitated for 1 h. Another solution was made by dissolving 0.633 g of the prepared ZnO NPs and 0.04261 g of Yb(NO_3_)_2_•5H_2_O in 45 mL of DI and stirring for 1 h at 70  °C. After that, the mixture was then added to the h‐BN solution and agitated for 25 min to create a homogenous mixture. Afterward, NaOH (0.1 m) solution was added dropwise until pH reached 9.5. The mixture was subsequently moved into a Teflon autoclaved reactor (100 mL) for further reaction at 200 °C for 6 h. The precipitates were repeatedly cleaned through the centrifugation method with DI water and absolute ethanol once the autoclave cooled to room temperature. The yellowish‐white‐colored residue was collected and left overnight in an oven to dry at 80 °C. Finally, the precipitate was calcined at 600°C for 4 h (ramping rate 3 °C min^−1^) and noted as YbZnO@h‐BN nanocomposites.

### 2.5 Fabrication of YbZnO@h‐BN/GCE Working Electrodes

The alumina slurry with a size of 1.0, 0.3, and 0.05 μm was used to thoroughly clean the GCE and ultrasonicated for 15 min with ethanol and DI water and subsequently dried at ambient temperature. About 2 mg of YbZnO@h‐BN nanocomposite was dispersed in DI water (1 mL) and ultrasonicated for 30 min. Then, ≈8  μL of the YbZnO@h‐BN suspension was then drop cast onto the well‐polished electrode and left to dry at ambient conditions. Finally, the electrochemical studies were conducted using the YbZnO@h‐BN/GCE modified electrode. Similar procedure was followed for ZnO/GCE, YbZnO/GCE, Ybh‐BN/GCE, and ZnOh‐BN/GCE WEs.

## 3 Results and Discussion

### 3.1 Material Characterization

**Figure 1:**
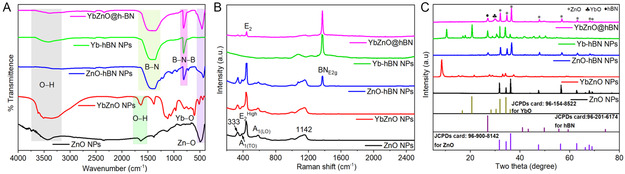
The FTIR spectrum, A) Raman spectrum, and B) and XRD pattern C) of ZnO NPs, YbZnO NPs, ZnO‐h‐BN NPs, Yb‐h‐BN NPs, and YbZnO@h‐BN nanocomposite.

The molecular interaction and functional groups of the synthesized ZnO NPs, YbZnO NPs, ZnO‐h‐BN NPs, Yb‐h‐BN NPs, and YbZnO@h‐BN nanocomposite were examined using FTIR spectroscopy (**Figure** **1**A). The ZnO FTIR spectrum (black line) shows the distinctive band between 400 cm^−1^ and 600 cm^−1^ from the Zn–O vibration mode. Furthermore, the O–H stretching vibrations from the free water molecules owing to the adsorbed moisture on the ZnO NPs surface are represented by the broad peak at 3450 cm^−1^.^[^ ^40^ ^]^ The YbZnO NPs spectrum (red line) shows the stretching bands at 506 cm^−1^ responsible for Zn**–**O bending vibration, while the band at 608 cm^−1^ was assigned to Yb–O stretching.^[^ ^41^ ^]^ The broad and sharp peaks at 1636 cm^−1^ and 3398 cm^−1^ are linked to the O–H stretching vibrations and the H–O–H bending vibration, respectively.^[^ ^42^ ^]^ For ZnO–BN NPs (blue line) and Yb–BN NPs (green line), two noticeable distinctive h‐BN peaks at 808 cm^−1^ and 1392 cm^−1^ were observed and were assigned to the out‐of‐plane B–N–B bending vibration (*A*_2u_) and in‐plane B–N stretching vibration (*E*_1g_), respectively.^[^ ^43^ ^]^ The characteristic bands of YbZnO NPs and h‐BN were clearly visible in the FTIR spectrum of the YbZnO@h‐BN (pink line), which signifies the successful synthesis of the nanocomposite.

The structural features of the prepared nanomaterials were further characterized using Raman spectroscopy, as depicted in Figure 1B. Raman spectrum of pure ZnO NPs and YbZnO NPs revealed five peaks that are detectable at the Raman shift of 329, 383, 436, 580, and 1149 cm^−1^, respectively. The most intense peak at 436 cm^−1^, which is the characteristic peak of ZnO (*E*_2_ high peak) and corresponds to oxygen atom vibration further supports the highly ordered crystallinity of the wurtzite structure.^[^ ^44^ ^]^ The *A1* (TO), *E1* (TO), and *A1* (LO) modes of ZnO are represented by the peaks at 329 cm^−1^, 383 cm^−1^, and 580 cm^−1^, respectively. Finally, the peak appearing at 1149 cm^−1^ can be assigned to the second‐order Raman processes.^[^ ^45^ ^]^ For ZnO‐h‐BN NPS and Yb‐h‐BN NPS spectra, the h‐BN characteristic peak (*E*_2g_ mode) associated with the in‐plane B–N stretching vibrations was confirmed by the predominant peak at 1373 cm^−1^.^[^ ^46^ ^]^ Interestingly, the YbZnO@h‐BN nanocomposite showed all these active Raman bands, indicating that YbZnO NPs have been successfully integrated into the h‐BN. However, a slight variation in Raman vibrational modes on the *E*_2_ high peak was observed. This could be ascribed to the introduction of Yb ions into the ZnO lattice. Thus, these findings further support the FTIR results in Figure 1A.

The crystallinity of the as‐prepared samples was ascertained using XRD, as shown in Figure 1C. The ZnO NPs XRD pattern revealed the hexagonal wurtzite phase with lattice constant *a* = *b* = 3.2530 Å and *c* = 5.2070 Å, which belongs to the *P6*_3_ *mc* space group. This structure is further confirmed with the reference pattern (JCPDS no. 96‐900‐8142).^[^ ^47^ ^]^ Additionally, XRD displayed high‐intensity peaks for ZnO NPs, suggesting high degree of crystallinity. No additional characteristic peaks were observed, confirming a pure ZnO phase. In the case of YbZnO NPs XRD pattern, a new additional peak emerged at 8.38°, 15.14°, 21.55°, 27.85°, and 29.99°, corresponding to the cubic Yb_2_O_3_ structural phase (JCPDF 27‐2374). This could be attributed to the difficulty in incorporating Yb ions into the ZnO lattice due to differences in their Shannon ionic radii: Yb^3+^ (0.89 Å) and Zn^2+^ (0.74 Å).^[^ ^48^, ^49^ ^]^ Albeit the characteristic peaks arise from the pure ZnO NPs, the XRD pattern for ZnO‐h‐BN NPs revealed a new characteristic signature of h‐BN at 26.79°, corresponding to the (002) plane matching the indexed pattern (JCPDF 96‐201‐6174).^[^ ^50^ ^]^ For the Yb‐h‐BN NPs, various sharp peaks were observed that matched well with those of the cubic C‐type Yb_2_O_3_ (JCPDF 98‐064‐7665, 96‐154‐8522), together with the distinctive peak for h‐BN.^[^ ^51^ ^]^ XRD patterns of YbZnO@h‐BN nanocomposites displayed the prominent characteristic features of zinc oxide crystal structure with fine crystallinity. In addition, the existence of additional diffraction patterns from Yb_2_O_3_ and h‐BN was observed, suggesting the mixed phase in the crystalline structure of the nanocomposite. However, the peak intensity of the typical diffraction pattern of h‐BN at 26.79° on the YbZnO@h‐BN was reduced, which could be ascribed to the decoration of YbZnO NPs. Furthermore, the most intense peak was the (101) plane corresponding to the ZnO structure with a slight shift toward the higher 2θ values. This could be explained by ytterbium doping, in which the Yb ions are randomly dispersed, resulting in the wurtzite crystal lattice anisotropic decrease and subsequent lattice deformation. The intrinsic strain on the ZnO crystal lattice caused by dopant atoms resulted in the notable broadening of diffraction peaks that are typical of the zinc oxide crystal structure in YbZnO@h‐BN. The XRD patterns of the prepared materials further support the results obtained from the Raman spectroscopy and FTIR in Figure 1A,B.

**Figure 2:**
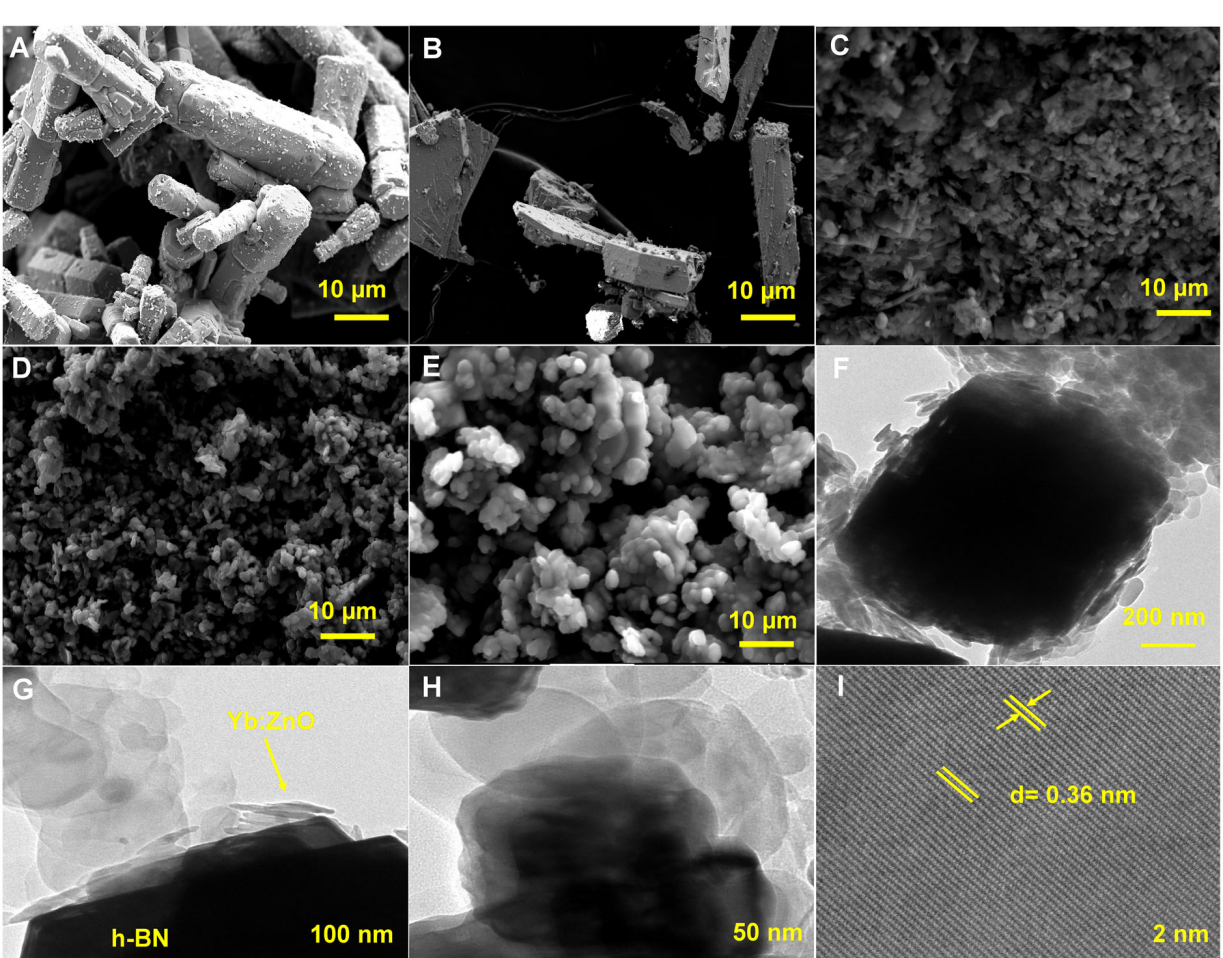
SEM image of A) ZnO NPs, B) YbZnO NPs, C) ZnO‐h‐BN NPs, D) Yb‐h‐BN NPs, and E) YbZnO@h‐BN nanocomposite. TEM images of YbZnO@h‐BN at different magnifications F–H), and I) the HAADF–STEM image of YbZnO@h‐BN nanocomposite.

The FESEM and TEM were used to examine the morphology of the synthesized materials as represented in **Figure** **2**. The top‐view image of pristine ZnO NPs displayed nonuniform microrod‐like morphology as depicted in Figure 2A. For YbZnO NPs (Figure 2B), it can be inferred that the rod‐like morphology was preserved for ZnO NPs with minor changes in morphological shape, owing to the addition of ytterbium ions in the ZnO structure. The ZnO‐h‐BN NPs revealed a clear aggregated microspherical particle with a rod‐like structure (Figure 2C), while the Yb‐h‐BN NPs showed small particles with clear agglomeration (Figure 2D). The YbZnO@h‐BN nanocomposite displayed microspherical particles that are stacked on top of each other (Figure 2E). TEM analysis confirmed the formation of the nanocomposite with aggregated large sheets decorated with small spherical and rod‐like particles (Figure 2 F–H), as observed in the SEM image. The HAADF–STEM image (Figure 2I) of the YbZnO@h‐BN nanocomposite lattice fringes showed a high degree of crystallinity with an approximate interplanar d‐spacing of 0.36 nm, which corresponds to the (002) planes of h‐BN. This suggests that the composite growth is in the [002] direction. EDX analyses were conducted to determine the element composition of the YbZnO@h‐BN nanocomposite, revealing the presence of all the constituent elements as shown in Figure S1A‐H, Supporting Information. Therefore, the earlier results confirm the successful synthesis of YbZnO@h‐BN nanosphere.

**Figure 3:**
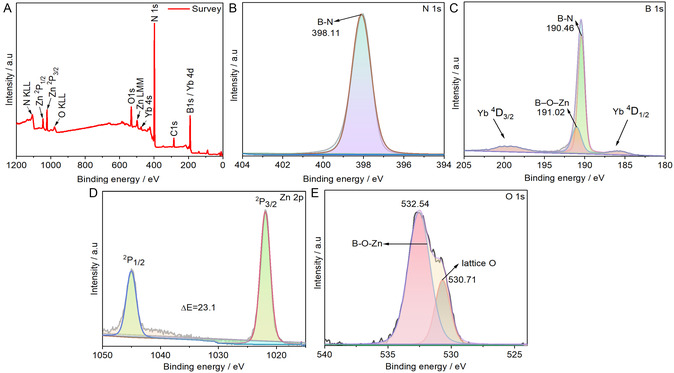
XPS A) survey, B) N 1s spectrum, C) B 1s spectrum, D) Zn 2p spectrum, and E) O 1s spectrum of the YbZnO@h‐BN nanocomposite.

To further interrogate the chemical states and surface composition of the YbZnO@h‐BN nanocomposite, XPS was employed. **Figure** **3**A displays the survey spectrum of the YbZnO@h‐BN composite, revealing the presence of B 1s, N 1s, C 1s, O 1s, Yb 4 d, and Zn 2p as the major elements present in the samples at 190.15, 398.63, 284.26, 531.60, 185.36, and 1022.16 eV, and substantiates the existence of the anticipated constituents without impurities. However, there is evidence of residual amounts of carbon, which could not be avoided due to the exposure of the sample to the ambient conditions. The core electron N 1s spectrum (Figure 3B) revealed one line centered at 398.11 eV, which corresponds to the N—B bond from the h‐BN structure.^[^ ^52^ ^]^ Figure 3C shows the high‐resolution spectrum of B 1s with two distinct lines centered at 190.46 eV and 191.02 eV, which is ascribed to the B—N bond from h‐BN and B—O—Zn bond, respectively.^[^ ^53^ ^]^ The XPS spectra of Zn 2p display a single binding environment as represented by Figure 3D. Due to the combined effects of both orbital and spin motion of electrons in an atom, the 2p energy level splits under spin–orbit coupling.^[^ ^54^ ^]^ As a result, the XPS spectrum showed two distinct lines centered at 1021.89 eV and 1044.99 eV that were assigned to Zn ^2^P_3/2_ and ^2^P_1/2_ levels, respectively, suggesting that the Zn atoms on the YbZnO@h‐BN nanocomposite exist in Zn^2+^ oxidation state and are in a tetrahedral coordination environment based on the binding energy separation (˜23.1 eV) in the ^2^P_3/2_ and ^2^P_1/2_ lines.^[^ ^55^ ^]^ The high‐resolution oxygen spectra for O 1s were deconvoluted into two lines at 530.701 eV and 532.54 eV (Figure 3 E). The 530.70 eV line can be assigned to the lattice oxygen O^2−^ ions (O_L_) present in YbZnO@h‐BN nanocomposite crystals, whereas the 532.53 eV line corresponds to the B—O—Zn chemical bond. These two bonds also show that ZnO NPs is successfully attached to h‐BN. The Yb 4 d XPS could not be fitted as the B 1s lines overlap with the Yb 4d lines, suppressing the lower intensity signal of Yb 4 d. The Yb ions were detected in EDX analysis (Figure S1H, Supporting Information), and the XPS survey spectrum shows that Yb is present in the nanocomposite.

### 3.2 Electrochemical Investigation

**Figure 4:**
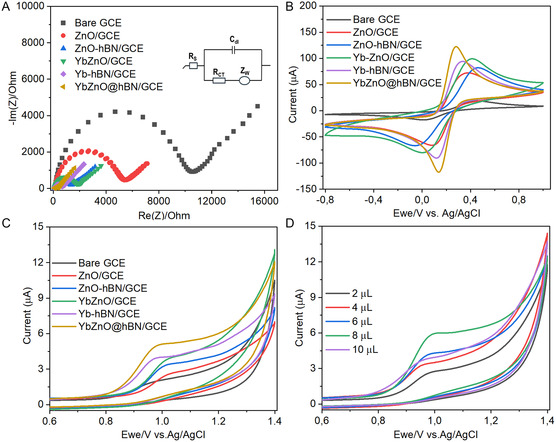
Electrochemical characterization of the modified and unmodified electrodes A) Nyquist plot, B) CV response of bare GCE, ZnO/GCE, ZnO‐h‐BN/GCE, YbZnO/GCE, Yb‐h‐BN/GCE, and YbZnO@h‐BN/GCE at 50 mV s^−1^ in 5 mM [Fe(CN)_6_]^3−/4−^ containing 0.1 M KCl C) CV studies of bare GCE, ZnO/GCE, ZnO‐h‐BN/GCE, YbZnO/GCE, Yb‐h‐BN/GCE, and YbZnO@h‐BN/GCE and D) CV recorded at different loading amounts of YbZnO@h‐BN/GCE in 0.1 M PBS containing 50 μM CIP at 50 mV s^−1^.

EIS was utilized to assess the electrocatalytic property of the WEs in 5 mM [Fe(CN)_6_]^3−/4−^ electrolyte comprising 0.1 M KCl. **Figure** **4**A presents a Nyquist plot of bare GCE, ZnO NPs/GCE, ZnO‐h‐BN NPs/GCE, YbZnO NPs/GCE, Yb‐h‐BN NPs/GCE, and YbZnO@h‐BN/GCE. It was worth noting that the bare GCE showed a higher *R*_ct_ value (11 030 Ω) and a wide semicircle diameter, which could be ascribed to its poor conductivity. However, the modification of the bare GCE with ZnO NPs leads to a decrease in semicircle diameter with a reduced *R*_ct_ (5501 Ω). Interestingly, after modifying with ZnO‐h‐BN NPs, YbZnO NPs, and Yb‐h‐BN NPs, the diameter of the semicircle notably shortened with *R*_ct_ values of 1074 Ω, 1764 Ω, and 5502 Ω, respectively. The decrease in *R*_ct_ values is ascribed to the improved surface area and conductivity on the WEs surface. However, the YbZnO@h‐BN/GCE showed a smallest semicircle diameter and a low *R*_ct_ value (251.8 Ω), suggesting the enhanced conductivity. This improvement is attributed to synergistic interactions between the YbZnO NPs and the 2D h‐BN structure in the composite, which enhances the electrode interface conductivity and facilitates mass transport of the [Fe(CN)_6_]^3−/4−^ ion. Therefore, the YbZnO@h‐BN/GCE electrode demonstrated rapid movement of electrons along with low charge transfer resistance, making it a suitable electrocatalyst for sensor development.

CV curves were recorded for the modified and unmodified electrodes in 5 mM [Fe(CN)_6_]^3−/4−^ comprising 0.1 m KCl and the sweep rate of 50 mV s^−1^ as illustrated in Figure 4B. All the CV curves displayed a quasi‐reversible [Fe(CN)_6_]^3−/4−^ redox couple.^[^ ^56^ ^]^ Moreover, the modified GCE showed a notable enhancement in redox peak current relative to the unmodified. The oxidation peak currents for the bare GCE, ZnO NPs/GCE, ZnO‐h‐BN NPs/GCE, YbZnO NPs/GCE, Yb‐h‐BN NPs/GCE, and YbZnO@h‐BN/GCE were 19.85 μA, 72.74 μA, 82.14 μA, 99.47 μA, 94.23 μA, and 121.96 μA, respectively. Furthermore, the difference in peak potential separations (Δ*E*_p_) was calculated and found to be +0.45 V, +0.28 V, +0.40 V, +0.32 V, +0.21 V, and +0.14 V versus Ag/AgCl, respectively. All future potentials are referenced to Ag/AgCl. Among all modified electrodes, the YbZnO@h‐BN/GCE electrode established the lowest Δ*E*_p_ (+0.14 V) and the highest oxidation peak current (121.73 μA), which indicates superior electrical conductivity and strong synergetic interaction, thereby improving its electrochemical activity. These CV curves corroborate the finding from EIS (Figure 4A), demonstrating that the h‐BN contributed to the surface area enhancement of the YbZnO‐modified electrode along with its conductivity. Therefore, for further electrochemical investigation, the YbZnO@h‐BN‐modified electrode was utilized.

### 3.3 The Electroactive Surface Area Determination

To determine the electroactive surface area (EASA) of the modified and unmodified electrodes, CV curves were recorded at various sweep rates (1 to 1000 mV s^−1^) in 5 mM [Fe(CN)_6_]^3−/4−^ containing 0.1 M KCl, as shown in Figure S2A–F, Supporting Information. It was noticed that increasing the sweep rate led to an increase in peak current, along with peak potential shift, suggesting the diffusion‐controlled process. Furthermore, the plot between the square root of the sweep rate (*v* ^1/2^) and peak current (*i*_p_) showed a good linear relationship, as illustrated in Figure S2A–F, Supporting Information. However, a worthwhile note was that the CV curves revealed pairs of quasi‐redox peaks as the sweep rate increased. Although the system exhibits the quasi‐reversible behavior, the Randles–Sevcik equation was used as an approximate method to estimate the EASA, as it offers a useful basis for comparison across different electrodes. Using the slope of the linear curve between anodic peak current and square root of the sweep rate, the EASA of the modified and unmodified electrodes was calculated using Equation (1)

(1) $$i_{p}=(2.69\;\times \;10^{5})Cn^{3/2}AD^{1/2}v^{1/2}$$

where *i*_p_ represents the peak current (A), *C* represents the concentration of the electroactive species (mol cm^3^), *n* is the number of electrons in a reaction, *A* is the electrochemical surface area (cm^2^), *D* is the diffusion coefficient of the [Fe(CN)_6_]^3−/4−^ redox probe, and *ν* is the sweep rate (V/s). Based on the results, the calculated EASA for the bare GCE, ZnO/GCE, ZnO‐h‐BN/GCE, YbZnO/GCE, Yb‐h‐BN/GCE, and YbZnO@h‐BN/GCE were 0.032, 0.034, 0.039, 0.042, 0.049, and 0.068 cm^2^, respectively.

### 3.4 Electrochemical Sensing of CIP Using Different Modified Electrodes

The piperazine ring at the seven positions in the CIP structure highlights its susceptibility to undergo oxidation and viability of direct assessment through electrochemical methods. Therefore, CV was exploited to examine the electrochemical behavior of 50 μM CIP at various WEs, including bare GCE, ZnO NPs/GCE, ZnO‐h‐BN NPs/GCE, YbZnO NPs/GCE, Yb‐h‐BN NPs/GCE, and YbZnO@h‐BN/GCE within the potential range of +0.6 V to +1.4 V in (0.1 M PBS; pH = 7.4) at the sweep rate of 50 mV s^−1^, as shown in Figure 4C. The bare GCE had a low peak current response for CIP at +0.99 V (*i*_pa_ 1.87 μA), suggesting slow electron transfer toward CIP oxidation.^[^ ^57^ ^]^ The corresponding current density was calculated to be 58.53 μA cm^−2^, which further supports the limited electrochemical activity of the unmodified electrode. However, upon the modification of the electrode with ZnO NPs/GCE, ZnO‐h‐BN NPs/GCE, YbZnO NPs/GCE, and Yb‐h‐BN NPs/GCE, a significantly enhanced peak current was noticed, owing to the improved electrocatalytic activity and larger effective surface areas.^[^ ^58^ ^]^ This enhancement was also evident in their increased current densities measured as follows: 59.86, 68.44, 77.95, and 83.42 μA cm^−2^, respectively. Among all, the YbZnO@h‐BN/GCE exhibited highest peak current response (*i*_pa_ = 5.84 μA) and current density (85.88 μA cm^−2^), with a minor shift in peak potential, highlighting its superior electron transfer efficiency and catalytic performance. Based on these findings, the YbZnO@h‐BN‐modified electrode demonstrated considerable attraction to CIP. This may be explained by the strong interaction between the vacant active sites of zinc ions in YbZnO@h‐BN, which quickly coordinate with the CIP molecular structure using the zinc ions’ interactive center in YbZnO@h‐BN. Due to the increased surface area, high conductivity, and synergistic interaction, the mass transport and electron transfer become rapid, thereby promoting the availability of electroactive sites for CIP detection.

### 3.5 Electrocatalyst Loading Volume

The amount of electrocatalyst deposited on the electrode surface may affect the sensor performance toward a target analyte. Hence, the influence of YbZnO@h‐BN catalyst loading suspension on the electrode surface was investigated from 2 to 10 μL. Figure 4D shows the CV response of YbZnO@h‐BN/GCE while varying catalyst loading volume in (0.1 M PBS, pH 7.4), comprising 50 μM CIP at a 50 mV s^−1^ sweep rate. The peak current increases with an increase in the YbZnO@h‐BN/GCE loading amount from 2 μL to 8 μL and reaches a highest current at ≈8 μL, which could be attributed to the creation of more available active sites for the electrochemical reaction.^[^ ^59^ ^]^ Further increase in YbZnO@h‐BN/GCE load to 10 μL resulted to the decline in peak current response. This can be explained by the formation of a dense layer on the surface of electrode at high catalyst loading, which could possibly elevate charge transfer resistance, thereby hindering movement of electrons.^[^ ^13^ ^]^ Therefore, 8 μL were selected as an ideal volume for the subsequent analysis.

### 3.6 Effect of Different pH

**Figure 5:**
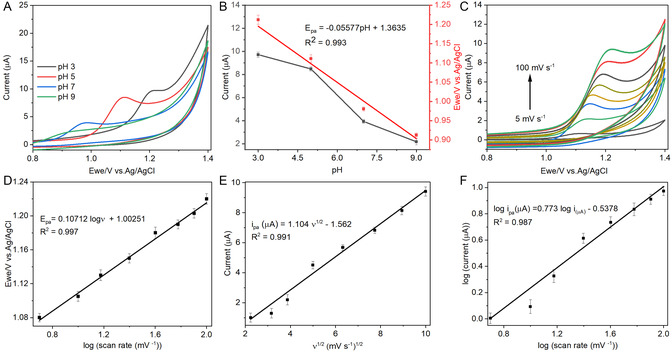
CV curves of YbZnO@h‐BN/GCE A) while varying pH (3.0 to 9.0) at the sweep rate (50 mV s^−1^), B) linear plot of pH versus current and peak potential, C) while varying sweep rate (5 to 100 mV s^−1^), D) linear plot of *E*_pa_ versus log *v*, E) linear plot of *i*_pa_ and *v* ^1/2^, and F) linear plot of log *i*_pa_ versus log *v*, in 0.1 M PBS containing 50 μM.

**Scheme 2:**
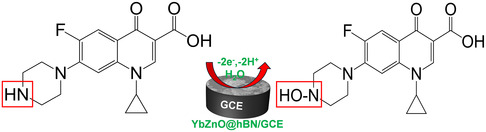
A possible mechanistic pathway for the CIP electrooxidation on the YbZnO@h‐BN/GCE‐modified electrode.

The influence of different pH (3.0 to 9.0) on the electrochemical efficiency of YbZnO@h‐BN/GCE sensor was assessed in 0.1 M PBS solution comprising 50 μM CIP, and the voltammograms are recorded in **Figure** **5**A. A progressive decline in the anodic peak current of CIP was noticed as the pH increased, accompanied by the negative potential shift, which signifies the participation of protons (^+^H) during the electro‐oxidation of CIP. The plot of pH and peak current is depicted in Figure 5B. The optimal choice for pH was based on the peak current and its profile, which express the kinetics of electron transfer, and pH 5.0 was selected as the optimum for further investigation. The plot of pH against peak potential (*E*_pa_) was established and displayed linear dependency with regression equation *E*_pa_ (V) = −0.05334 pH + 1.4735 (*R* ^*2*^ = 0.995) (Figure 5B), which further supports the proton involvement in electrochemical determination of CIP. Furthermore, the value of the slope of this curve can be employed to predict the number of protons and electrons participating in the electrochemical reaction. Hence, these findings corroborate with the existing value of the Nernst equation (–0.0591 V pH^−1^), suggesting the equivalent number of protons and electrons transferred toward CIP oxidation on the YbZnO@h‐BN/GCE.^[^ ^60^ ^]^ Due to the secondary amine on the CIP chemical structure, a potent electron‐withdrawing group, therefore, it can be reasoned that its oxidation to generate the hydroxylamine derivative may be the driving force allowing CIP to undergo the oxidation process. These results enabled conjecture regarding the possible mechanistic pathway for the electrooxidation of CIP at the YbZnO@h‐BN/GCE interface, **Scheme** **2**.

### 3.7 Effect of Sweep Rate

To attain more evidence on the kinetics of the YbZnO@h‐BN/GCE electrode, the influence of sweep rate (*v*) (5 to 100 mV s^−1^) was examined using CV in PBS solution (0.1 M, pH 5.0) comprising 50 μM CIP (Figure 5C). It was worth noting that both the CIP current and potential were impacted by *ν*, and an increase in sweep rate resulted in the enhancement of anodic peak currents, together with a slight positive shift in peak potentials. According to the equation I *= Q/t* (where *I* represents current in A, *Q* is electric charge in C, and *t* denotes time in seconds), current and time are related through the rate of charge transfer. In CV, increasing the sweep rate lessens the time it takes to complete each cycle, thereby elevating the anodic peak current due to rapid electron transfer. Moreover, the positive shift in peak potentials indicates kinetic limitation, leading to a charge transfer overpotential needed to compensate for slower reaction at the surface of the electrode. Figure 5D depicts the linear plot between the logarithm of sweep rate (log *v*) against potential (*E*_pa_), and the corresponding equation *E*_pa_ (V) = 0.10712 log *v* + 1.00251, *R* ^*2*^ = 0.997. Furthermore, the plot of peak current against the square root of sweep rate displayed a straight‐line graph with regression, Equation (1) *i*_μA_ = 1.104 *v* ^1/2^ – 0.1562, *R* ^*2*^ = 0.991, suggesting a diffusion‐controlled mechanism governing the electrochemical oxidation of CIP on YbZnO@h‐BN/GCE (Figure 5E). Furthermore, a positive minor variation in peak potential as the sweep rate increases reflects the irreversibility of the electrochemical process. Interestingly, the plot of log current against log sweep rate was obtained and exhibits a linear relationship that aligns with the equation log *i*_pa_ (μA) = 0.773 log *v* (mV s^−1^) – 0.5378, *R* ^*2*^ = 0.987 (Figure 5F). This indicated that there were some CIP molecules which were being adsorbed on the YbZnO@h‐BN/GCE surface, owing to the π–π interactions, hydrogen bonding, and electrostatic interactions.

### 3.8 Electrochemical Evaluation of CIP at the YbZnO@h‐BN/GCE

**Figure 6:**
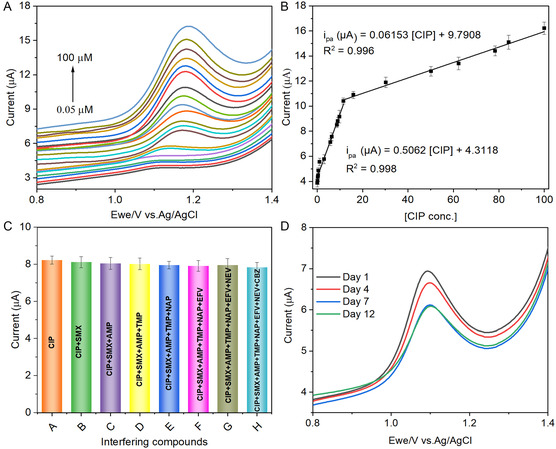
SWV response of the YbZnO@h‐BN/GCE electrode A) while varying CIP concentration (0.05 μM to 100 μM), B) the plot of concentration (CIP) versus current, C) bar graph of 10 μM CIP in the absence and presence of interfering species, and D) stability over 12 days in PBS (0.1 M, pH = 5.0) at the scan rate of 25 mV s^−1^.

The SWV technique was adopted to evaluate the YbZnO@h‐BN/GCE electrode on anodic peak current while varying CIP concentrations. **Figure** **6**A shows the SWV response of YbZnO@h‐BN/GCE sensors while varying concentrations of CIP from 0.05 μM to 100 μM. An increase in CIP concentrations caused a gradual increase in peak current, accompanied by a minor shift in peak potential. The plot of peak current (*i*_pa_) versus CIP concentration demonstrated a linear relationship with two distinct linear dynamic ranges from 0.05 to 10 μM and 10 to 100 μM, respectively. The corresponding regression equations were determined as *i*_μA_ = 0.5062 [CIP] + 4.3118, *R* ^*2*^ = 0.998, *i*_μA_ = 0.0653 [CIP] + 9.7908, and *R* ^*2*^ = 0.996, respectively (Figure 6B). LOD was estimated to be 0.059 μM based on the slope value of the lower dynamic range. These observations obviously demonstrate the effectiveness of the YbZnO@h‐BN/GCE sensor for the electrooxidation of CIP, offering a broad linear dynamic range and low detection limit. The efficiency of the YbZnO@h‐BN/GCE‐decorated electrode was further compared to the already existing sensor in terms of linear range and LOD (**Table** **1**). Herein, the proposed sensor exhibited a broad detection range in comparison to previous reports, enabling the determination of CIP at low concentrations. In addition to electrochemical sensing approaches, several other technologies have been developed for antibiotic detection, offering diverse sensing principles and performance characteristics. For example, in the study conducted by Dai et al. (2023), an optical fiber SPR biosensor with laser heterodyne feedback was developed for the detection of FQs.^[^ ^61^ ^]^ This method demonstrated high sensitivity and real‐time detection but typically requires complex surface functionalization and instrumentation. However, the YbZnO@h‐BN/GCE sensor is relatively simple to synthesize, with h‐BN proving the advantage of a large surface area, while the YbZnO NPs are environmentally friendly.

**Table 1:** Comparison studies of the YbZnO@h‐BN/GCE sensor with previously documented sensors for CIP detection.

Sensors	Method	Analyte	Linear range [μM]	LOD [μM]	Ref
Ru‐Cu‐TMA/GCE	DPV	CIP	2.5 to 100	0.329	[58]
MnO_2_/ZnO/GCE	DPV	CIP	0.5 to 120	0.21	[62]
GO/PEI/TiO2/aptamer/GCE	DPV	CIP	0.003 to 10	0.0007	[63]
Py‐BM‐CH_3_/ptE	LSV	CIP	0.01 to 200	2.75	[64]
rGO/PPR/GCE	DPV	CIP	0.05 to 400	0.002	[65]
V_2_O_5_/SPE	DPV	CIP	0.04 to 365	0.01	[66]
NH_2_–UiO‐66/RGO/GCE	ASV	CIP	0.02 to 1	0.00667	[67]
PDA‐MWCNTs/GCE	DPV	CIP	0.07 to 100	0.04	[68]
GO‐SPCE	DPV	CIP	1.0 to 8.0	0.30	[69]
f‐MWCNT‐coated GCE	SWV	CIP	5 to 100	0.16	[70]
ZnBC/GCE	DPV	CIP	0.08 to10	0.418	[71]
YbZnO@h‐BN/GCE	SWV	CIP	0.05 to 100	0.059	This work

### 3.9 Selectivity, Long‐Term Stability, and Reproducibility Studies

For real‐time applicability of an electrochemical sensor, selectivity, stability, and reproducibility are crucial factors for determining the suitability of target analytes in actual samples. Thus, the selectivity of the proposed sensor was evaluated in the presence of possible interfering compounds under optimized conditions by adding 10 μM CIP along with 100 μM of interfering species, including sulfamethoxazole (SMX), carbamazepine (CBZ), naproxen (NAP), efavirenz (EFV), trimethoprim (TMP), ampicillin (AMP), and nevirapine (Figure 6C). The result revealed no significant changes in Δ*i*_pa_ signals of CIP when exposed to these interfering species, suggesting an excellent selectivity of YbZnO@h‐BN/GCE toward CIP with RSD of less than 5.0%.

To evaluate the reliability of the YbZnO‐h‐BN/GCE‐modified electrode, the sensor was stored at room temperature for 12 days. SWV was used to examine the robustness of the YbZnO‐h‐BN/GCE every three days in PBS solution (0.1 m, pH = 5.0) comprising 10 μM CIP (Figure 6D). However, there was a noticeable change in CIP signal response from day 1 to day 7, which could be attributed to factors such as storage conditions, electrode surface degradation, or even the stability issue of the YbZnO@h‐BN composite material over time. Furthermore, the electrode remains unchanged after 7 days to 12 days and retains 88.7% of its original current response, indicating good stability of YbZnO‐h‐BN/GCE sensor.

To assess the reproducibility of the YbZnO‐h‐BN/GCE, SWV was performed in PBS solution (0.1 M, pH = 5.0), comprising 10 μM CIP using four different electrodes prepared with the same procedures (Figure S3, Supporting Information). The response current of the four‐electrode displayed the relative standard deviation (RSD) of 3.4%, which indicates good reproducibility of the sensor in determining CIP.

### 3.10 Real Samples Assessment

The SWV was used to investigate the feasibility of the YbZnO@h‐BN‐modified electrode for CIP determination in real wastewater samples and commercial CIP tablets under optimum conditions following the conventional addition approach. The water sample was collected from Umgeni wastewater treatment plant located in Kwazulu‐Natal Province, South Africa. The commercial CIP tablet was procured at the local pharmacy in Johannesburg, South Africa. The wastewater samples were first filtered with Whatman filter papers to ensure purity. This was then followed by correcting the pH to the optimal value (pH 5.0). The SWV response of the background scan was recorded before the addition of a known CIP concentration into water sample (Figure S4A, Supporting Information). Similarly, the commercially available tablet was dissolved in PBS (0.1 M, pH = 5.0) and diluted to the required concentration after being ground into fine powder, and SWV was employed to record the peak current (Figure S4B, Supporting Information). The results demonstrated satisfactory recovery values in wastewater sample and the tablet, as shown in **Table** **2**. The recovery values were determined and ranged from 94% to 105% and 95% to 112%, respectively. Furthermore, the RSD values in wastewater and commercial CIP tablets were less than 5% (n = 3) based on the three parallel measurements. These findings demonstrate that the YbZnO@h‐BN‐modified electrode was suitable for the determination of pharmaceutical products in real water samples.

**Table 2:** Determination of CIP in real wastewater samples and commercially available tablets on the YbZnO@h‐BN/GCE.

Sample	Added [μM]	Found [μM]	Recovery [%]	RSD [%]
^∗^WWTPs	1	1.05	105	3.45
	2	1.88	94	4.26
	3	3.06	102	1.52
CIP tablet	1	0.95	95	3.63
	2	2.23	112	7.69
	3	2.94	98	1.43

^∗^WWTPs–Wastewater treatment plant.

## 4 Conclusion

The YbZnO@h‐BN nanocomposite was successfully prepared through hydrothermal method. The XRD revealed the formation of mixed‐phase structure of YbZnO@h‐BN composite. In addition, the SEM, TEM, and FTIR showed that YbZnO NPs were effectively embedded on the bulk h‐BN. YbZnO@h‐BN/GCE‐modified electrode was effectively developed for CIP detection in an actual wastewater sample. Compared to the ZnO/GCE (2.04 μA), ZnOh‐BN/GCE (2.65 μA), YbZnO/GCE (3.27 μA), and Ybh‐BN/GCE (4.01 μA), the YbZnO@h‐BN/GCE showed the highest current response (5.84 μA). This improvement was attributed to the synergistic interaction between YbZnO NPs and h‐BN, which improves conductivity and EASAs of YbZnO@h‐BN/GCE. For CIP detection, the YbZnO@h‐BN/GCE sensor demonstrated a low detection limit (0.059 μM), a broad linear range (0.05 to 100 μM), and high sensitivity (7.4441 μA μM^−1^ cm^−2^). Furthermore, the proposed sensor demonstrated good stability, repeatability, and selectivity for CIP. The applicability of the YbZnO@h‐BN/GCE sensor displayed excellent recoveries of 94 to 105% in wastewater treatment plants (WWTPs) and 95% to 112% in commercial tablets, respectively. Therefore, the YbZnO@h‐BN/GCE‐modified electrode provides a fresh method for identifying CIP in wastewater for pharmaceutical analysis.

## Conflict of Interest

The authors declare no conflict of interest.

## Author Contributions

**Collen Nepfumbada**: Conceptualization, Methodology, Investigation, Data Curation, and Writing—Original Draft, Review & Editing. **Christopher P. Woodley**: Analysis, Methodology, Validation, and Writing—Review & Editing. **Bhekie B. Mamba**: Supervision, Resources, Validation, and Writing—Review & Editing. **Bart M. Bartlett**: Validation, Visualization, and Writing—Review & Editing. **Usisipho Feleni**: Supervision, Resources, Conceptualization, Validation, Visualization, Project Administration, and Writing—Review & Editing. All authors discussed the results, completed a final draft, and agreed to submit it.

## Supporting information

Supplementary Material

## Data Availability

The data that support the findings of this study are available from the corresponding author upon reasonable request.
