# Supplementary material for: Ytterbium–Zinc Oxide Embedded on Hexagonal Boron Nitride Nanocomposite for Electro‐Oxidation of Ciprofloxacin
Source: ChemistryOpen. 2025 Jun 26;14(11):e202500246. doi: 10.1002/open.202500246 (PMC12598802; doi:10.1002/open.202500246)
Supplement: Supplementary file 1 — Supplementary Material [file OPEN-14-e202500246-s001.pdf]

Ytterbium-Zinc Oxide Embedded on hexagonal Boron Nitride Nanocomposite for Electro-oxidation of Ciprofloxacin

Collen Nepfumbada[1*]; Christopher P. Woodley[2]; Bhekie B. Mamba[1]; Bart M. Bartlett[1,2]; Usisipho Feleni[1*]

[1]*College of Science, Engineering and Technology (CSET), University of South Africa (UNISA), Florida campus, 1709 Johannesburg, South Africa, Institute for nanotechnology and water sustainability (iNanoWS*

[2]*Department of Chemistry, University of Michigan, Ann Arbor, Michigan 48109-1055, United States.*

[*]*Corresponding author:* *feleni@unisa.ac.za*;18028896@mylife.unisa.ac.za

Table of Contents

**Figure S1**. (A) TEM image, (B) B, N, O, Zn, and Yb, (C) O, (D) Yb, (E) N, (F) B, (G) Zn EDX-TEM map images, and (H) SEM-EDX spectrum of YbZnO@hBN nanocomposite.	S2
**Figure S2:** CV response of the modified and unmodified electrodes and their corresponding linear regression line (A) bare GCE, (B) ZnO/GCE, (C) ZnO-h-BN/GCE, (D)YbZnO/GCE, (E)Yb-h-BN/GCE, and (F) YbZnO@h-BN/GCE at various sweep rate (1-1000 mV s^{-1}) in 5 mM $[Fe(CN)_6]^{3-/4-}$ containing 0.1 M KCl.	S3
**Figure S3**. SWV response of different YbZnO@h-BN/GC modified electrodes.	S3
**Figure S4**. SWV response of the YbZnO@h-BN/GCE sensor in real sample (A) WWTPs and (B) commercial tablet	S4

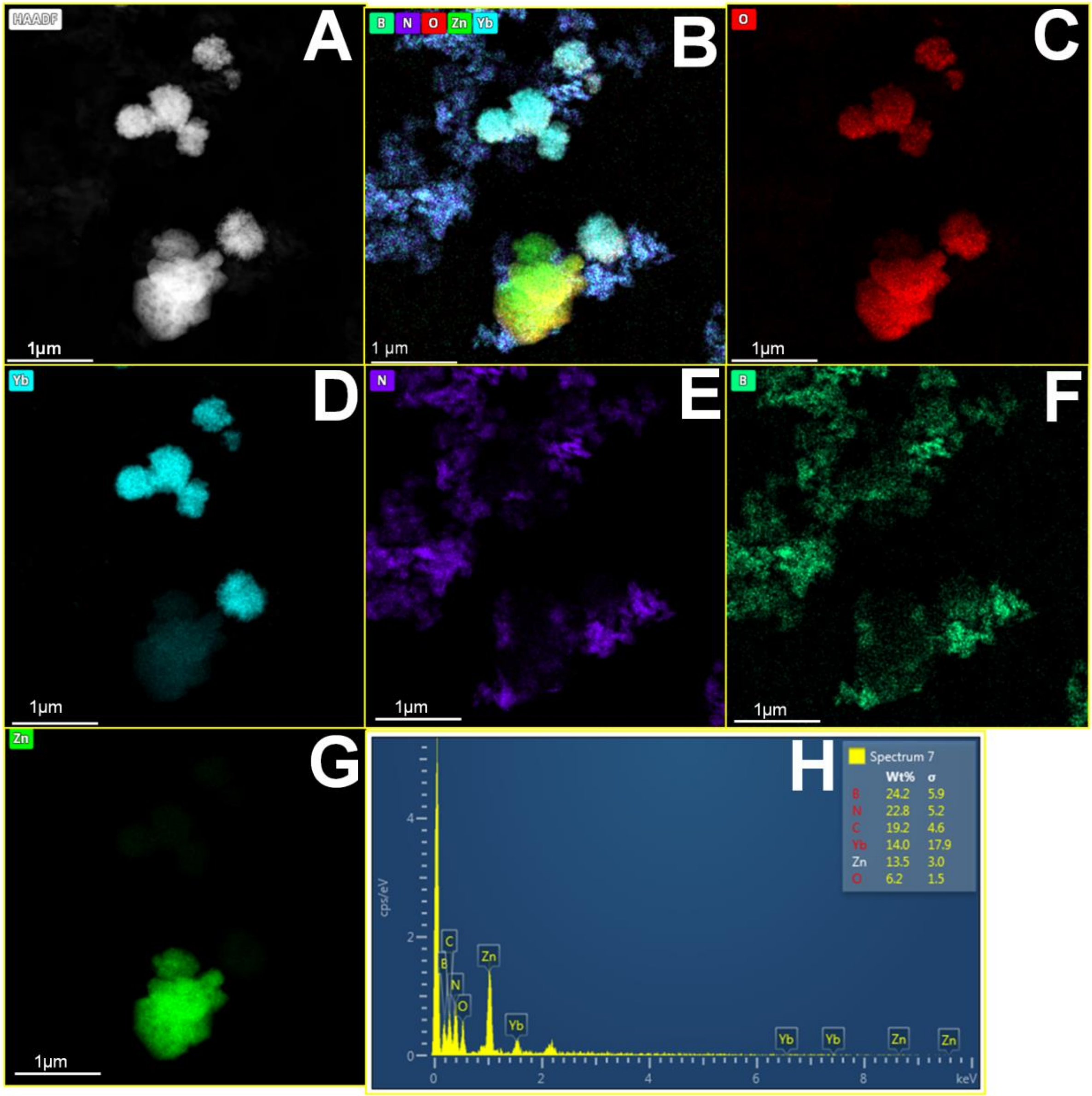

Figure S1: (A) TEM image, (B) B, N, O, Zn, and Yb overlay, (C) O, (D) Yb, (E) N, (F) B, (G) Zn EDX-TEM map images, and (H) SEM-EDX spectrum of YbZnO@h-BN nanocomposite.

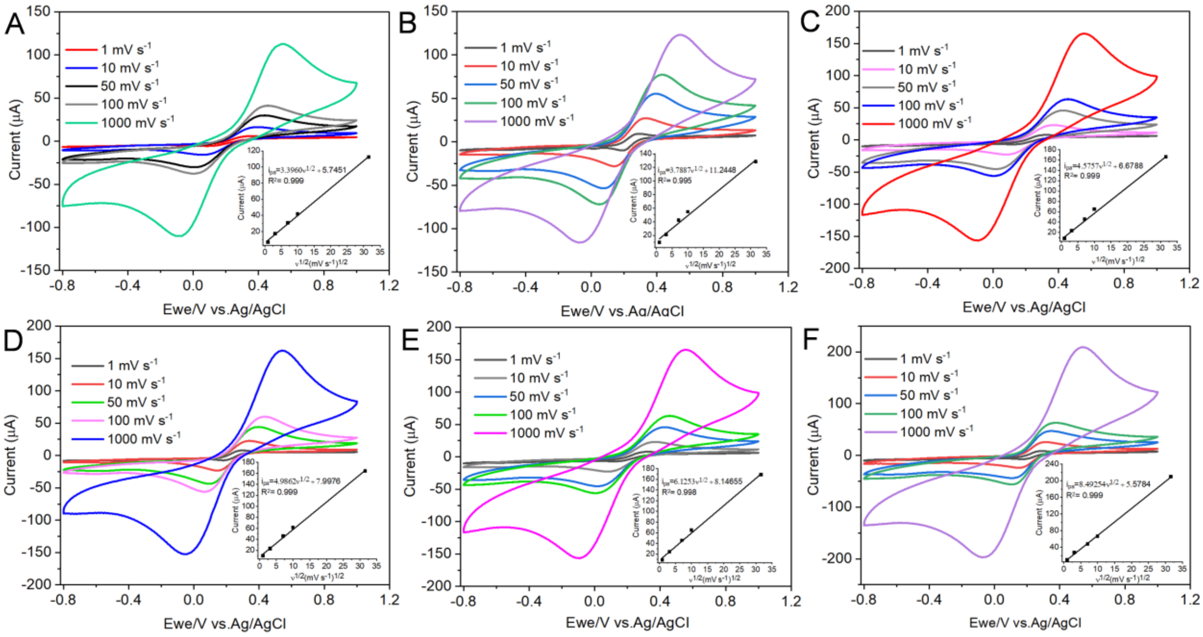

Figure S2: CV response of the modified and unmodified electrodes and their corresponding linear regression line (A) bare GCE, (B) ZnO/GCE, (C) ZnO-h-BN/GCE, (D)YbZnO/GCE, (E)Yb-h-BN/GCE, and (F) YbZnO@h-BN/GCE at various sweep rate (1-1000 mV s^{-1}) in 5 mM $[Fe(CN)_6]^{3-/4-}$ containing 0.1 M KCl.

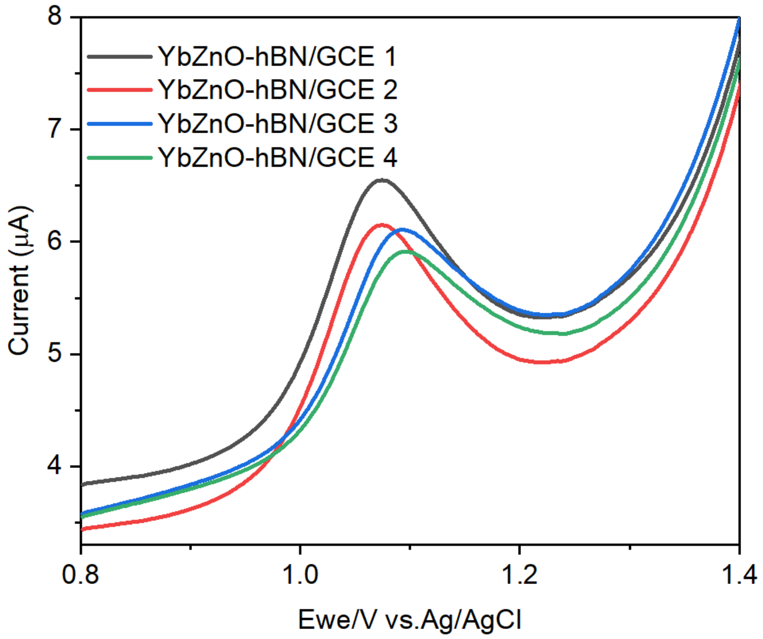

Figure S3: SWV response of different YbZnO@h-BN/GCE modified electrodes.

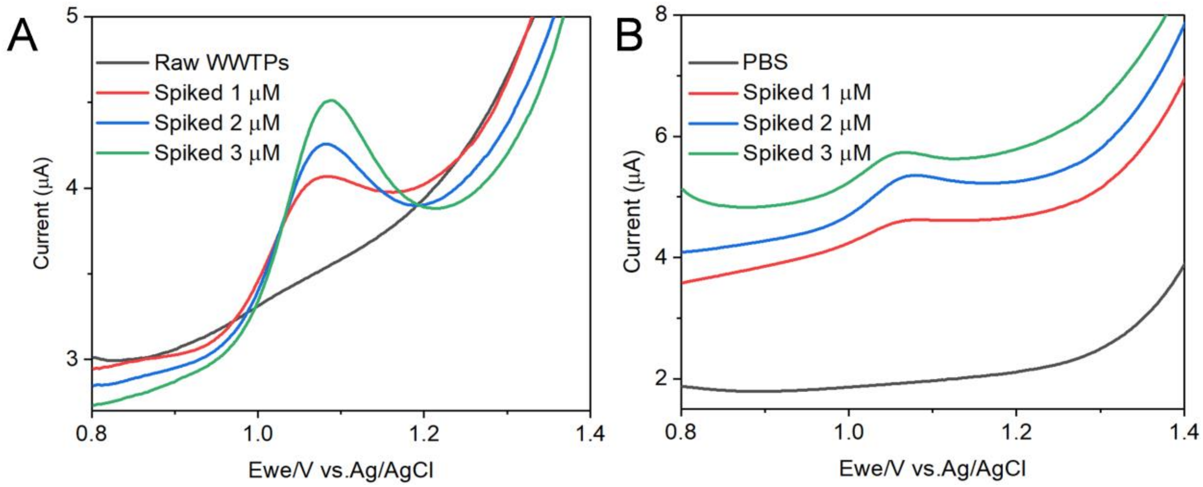

Figure S4: SWV response of the YbZnO@h-BN/GCE sensor in real sample (A) WWTPs and (B) commercial CIP tablet.
